# Aneurysmal bone cyst in thoracolumbar spine

**DOI:** 10.1259/bjrcr.20190133

**Published:** 2020-04-01

**Authors:** Alex Kiu, Tiffany Fung, Pranav Chowdhary, Sungmi Jung, Tom Powell, Mathieu Boily

**Affiliations:** 1 Department of Diagnostic Radiology, McGill University Health Center, MontrealQC, Canada; 2 Department of Pathology, McGill University Health Center, MontrealQC, Canada

## Abstract

Aneurysmal bone cysts (ABC) are rare, benign primary bone tumors. Although benign, they can be locally aggressive resulting in erosion of bone and surrounding tissues over time. In later stages, depending on the clinical urgency, immunotherapy or surgical resection remain treatment options. This report illustrates a case of a 32-year-old female who presented with chronic worsening low back pain without neurological deficits. Radiological imaging revealed a large destructive mass arising from the thoracic spine invading into the central canal, causing critical central stenosis and cord compression. Histopathology revealed ABC. This case highlights the importance of including ABCs and other ‘benign’/locally aggressive lesions in the differential of patients with insidious musculoskeletal complaints. This case also demonstrates that one can be neurologically asymptomatic despite having critical central canal stenosis and cord compression if the causative lesion is slow growing. Understanding this allows us to arrange for most appropriate management.

## Case presentation

A 32-year-old female who is 7 months post-partum presented with left-sided lower back pain that began during the second trimester of her pregnancy. The pain was originally attributed to her pregnancy but persisted post-partum. She described the pain as worse when supine with occasional radiation to the left gluteal area. She is otherwise healthy with no neurological deficits. The review of systems was negative and the patient did not report any systemic symptoms. On physical examination, the patient had no pain on palpation of the spine and normal symmetric strength and sensation as well as intact reflexes with normal gait patterns.

## Investigations and image findings

**Figure 1.**
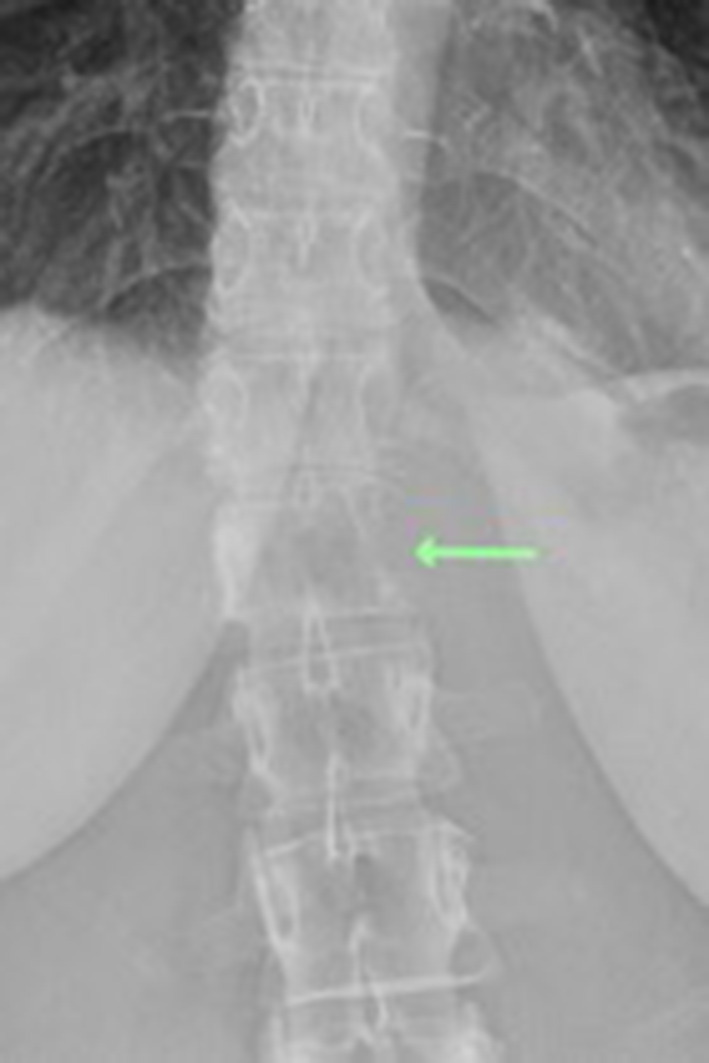
Plain radiograph demonstrating bony irregularity along the left border of T12 vertebral body and to a lesser extent the T11 level. Note there is mild curvature of the thoracolumbar junction convex to the left which may be secondary to pain.

A plain radiograph of the hip demonstrated bony irregularity along the left border of T12 vertebral body and to a lesser extent the T11 level (Figure 1).

**Figure 2.**
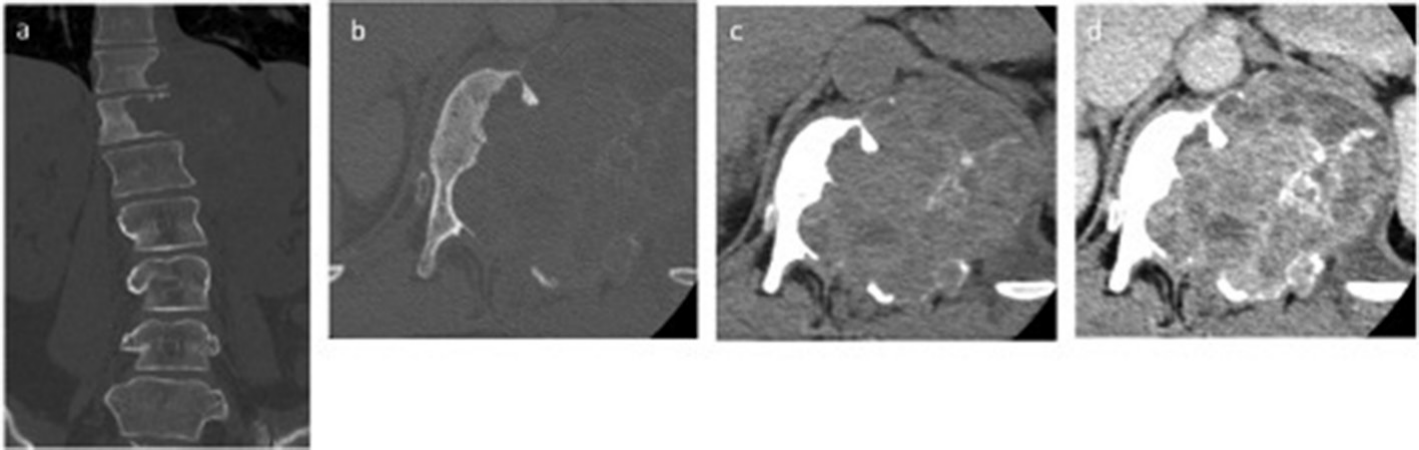
(a–d) (a, b) Non-contrast CT bone windows demonstrates bony destruction at T11 and T12 vertebral level with a large soft tissue mass adjacent to it. There is quite prominent bony destruction but there is well-circumscribed borders on closer inspection. Note fine bony septations in b. (c, d:) Pre- and post-contrast CT studies in soft tissue window demonstrating prominent heterogenous soft tissue enhancement.

CT of the lumbar spine was performed and revealed a large 10 cm irregular, destructive expansile soft tissue mass with its epicentre arising from the T11 and T12 vertebral body and left pedicle with body destruction of the left-sided posterior elements (Figure 2). The mass had cystic and soft tissue densities within, thin irregular osseous septations with prominent internal heterogenous enhancement. This mass invaded the central canal causing severe central canal stenosis at the T12 level, as well as invasion of the T11-T12, and T12-L1 exit neural foramina.

**Figure 3.**
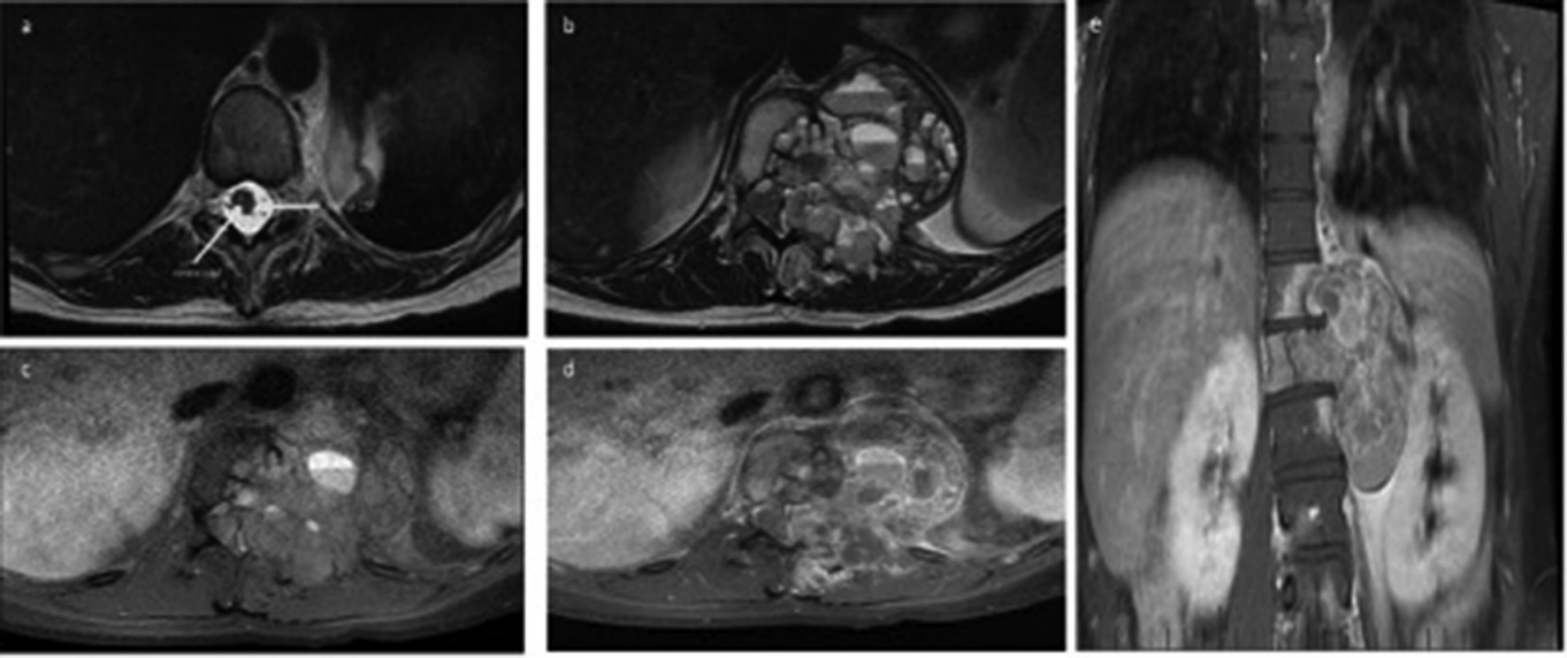
(a**–**e) A 32-year-old female presents with vague left-sided lower back pain.

MRI of the spine revealed the lesion to contain multiple fluid-fluid levels (Figure 3). It has well-circumscribed borders causing scalloping of the adjacent bony structures, suggesting a chronic locally aggressive pathology. It measured 8.1 × 7.4 × 9.8 cm (transverse x anteroposterior x craniocaudal). The lesion eroded into the central canal, causing significant central canal stenosis, displacing the cord with complete effacement of the surrounding cerebrospinal fluid (CSF). There was however no cord edema at this this level or the levels above and below. The lesion also extended to involve the proximal left psoas muscle causing compressive soft tissue edema and inflammatory changes.

The patient underwent ultrasound-guided biopsy, which was preferred over CT guidance for several reasons. Ultrasound guidance would reduce radiation exposure to the patient and could be performed more quickly and readily, thus reducing the time to diagnosis. While there is a theoretical risk of under sampling, the track of entry and target of biopsy was planned with sarcoma orthopedic surgeon to ensure optimal success.

The biopsy was performed via posterior approach, under sterile conditions and with local anesthesia. Biopsy specimens were obtained using an 11-gauge access and 13-gauge biopsy needle together with the powered needle driver. Three core samples were obtained and the specimens were sent to the pathology department for analysis.

An MRI of lumbar spine revealed a large expansile mass arising from T12 vertebral body. (a) Axial T2 sequence of a normal central canal (arrows demonstrating CSF space and normal spinal cord). (b) Axial T2 sequence demonstrates with internal heterogeneity with multiple fluid–fluid levels, invading into the central canal causing significant central canal stenosis, displacing the cord with complete effacement of the surrounding CSF but no cord edema. The borders of the lesion are however well circumscribed with scalloping of the adjacent bony structures, suggesting a chronic locally aggressive pathology. (c) Axial T1FS sequence demonstrates central areas of hematoma within some of the fluid–fluid components. (d) Axial T1FS post-contrast sequence demonstrates soft tissue heterogenous enhancement. Coronal T1FS post-contrast demonstrating the size of lesion and is well circumscribed.

## Differential diagnosis

The differential diagnosis for the CT-demonstrated destructive lesion would include but not be exclusive to primary and secondary malignancies (specifically vascular metastases such as renal cell, breast or melanoma) dependant on the age group. However, the clinical presentation of chronicity together with the imaging findings suggests a slow-growing and locally aggressive lesion such as a giant cell tumour, aneurysmal bone cyst, osteoblastomas or chrondroblastomas.

The differential diagnosis for the MR findings of a fluid–fluid level include but is not exclusive for an aneurysmal bone cysts (ABC) (either primary or secondary to a giant cell tumor) or more aggressive lesions such as telangiectatic osteosarcoma or hypervascular metastases such as renal cell or lung. ABCs and telangiectatic osteosarcomas share many imaging features but have very different prognoses. They are often difficult to distinguish in large and locally aggressive cases such as this and histopathological correlation is required to obtain a final diagnosis.

**Figure 4.**
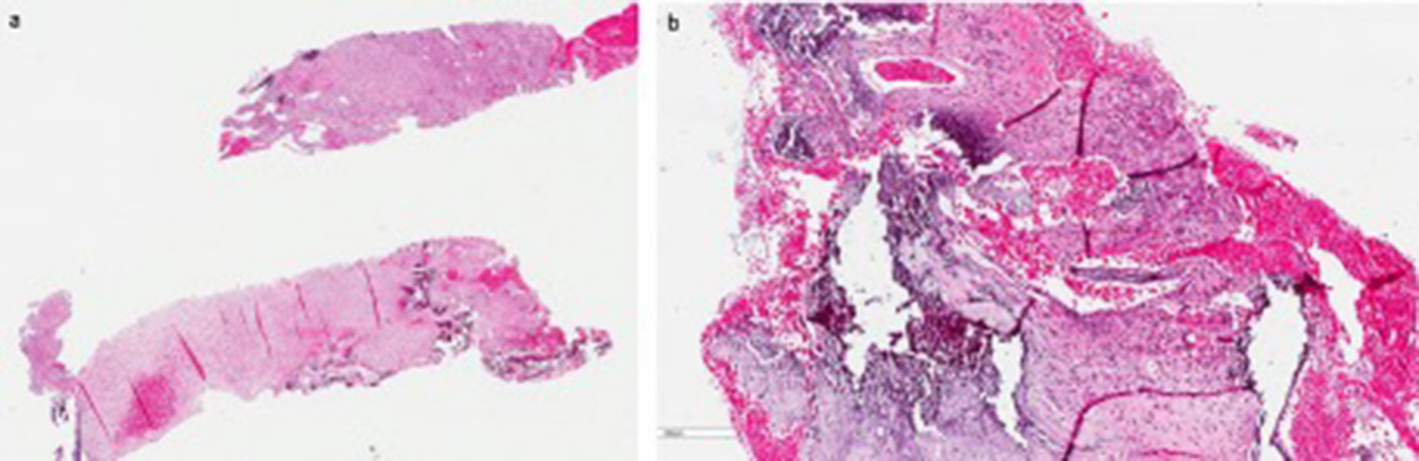
(a–b) (a) The biopsy shows multilocular cysts and solid fibrous tissue with hemorrhage. (b) The cysts are lined by fibroblastic spindle cells and loose fibrous tissue with scattered osteclastic giant cells, reactive woven bone formation and calcification.

The patient underwent an ultrasound-guided biopsy. Histological examination revealed a primary aneurysmal bone cyst (Figure 4).

## Treatment

In view of the imaging findings of severe central canal stenosis, orthopedic spinal team and oncology team were consulted urgently. The patient’s case was discussed at an interdisciplinary tumor board meeting which recommended that the patient be started on denosumab. Denosumab is a human monoclonal antibody that binds to the human RANK ligand (RANKL) on the surface of osteoclasts and their precursors, inhibiting osteoclast formation, function and survival, leading to decreased bone loss and destruction.^1^ Plan for re-evaluation in a few months to assess the need for surgery to stabilize the spine. The patient was started on Denosumab monthly subcutaneous injections. She was also started on calcium and vitamin D supplements.

## Outcome and follow-up

**Figure 5.**
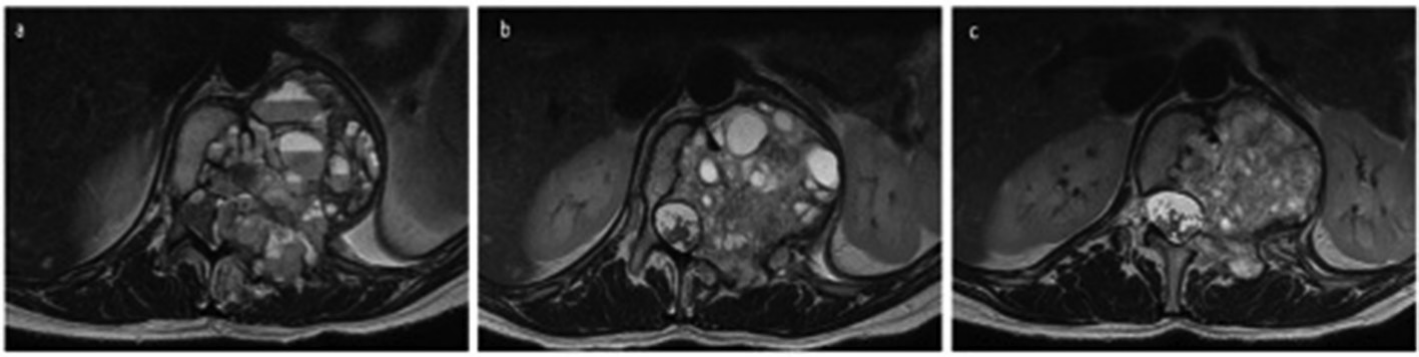
(a–c) MRI of patient pre-treatment, 4 months post-treatment and 9 months post-treatment respectively in sequential order. (b, c:) Axial T2 sequence at T12 level demonstrates interval decrease in size of lesion with little or no epidural involvement. There is now normal CSF spaces with no compression against the cord. CSF, cerebrospinal fluid fluid.

A repeat MRI 4 and 9 months later showed interval shrinkage of the entire lesion with significant improvement of the pressure mass effect on the central canal, with only mild central canal stenosis (Figure 5). The patient tolerated the treatment well and no longer experiences any pain. She is still actively followed by both the orthopedic spine and oncology teams. The current management plan is to continue with Denosumab and proceed with stabilization surgery after a few months upon re-evaluation by the orthopedic spine and oncology teams.

## Discussion

ABC are rare, benign primary bone tumors that can be fast-growing and locally destructive.^2^ They represent approximately 1.4% of primary bone tumors. Among primary bone tumors, 3–30% of ABC cases involve the vertebral column, particularly the posterior elements.^2,3^ Prevalence of ABC varies within the vertebral column, with the cervical spine being involved in 30–40% of cases, the thoracic spine in 25–50% and the lumbar spine being most common in 40–45% of cases.^3^

Other areas affected include the mandible, clavicle, feet, and fingers.^4^ They occur predominantly in the pediatric population but can occur in adults, often in the first two decades of life and with a female predilection.^2^

They are expansile entities that can cause significant osteolytic destruction of bone and surrounding tissue.^2^ It can result in large lesions affecting the spine leading to significant morbidity. While there are discussions of ABCs in the literature, many of them involve ABCs of the mandible and literature about ABC of the thoracic spine are limited.^5^

Patients with an ABC often present with pain in the affected area along with neurological deficits. Paresthesia, paresis and abnormal gait are common neurological manifestations when the spine is involved.^6,7^ However, we present a case of a female with non-specific back pain and no other significant neurological symptoms. She had no evidence of pathological fracture or progressive paraplegia which would raise the usual red flags for an underlying aggressive process. It is therefore important to consider this rare entity among the list of differential diagnoses for nonspecific, chronic back pain, especially among younger patients.

It is important to proceed with imaging modalities to make the proper diagnosis as ABCs have characteristic features. Its erosive process can be represented as destruction of the facet joints along the vertebral column. A plain film of the spine or the area of interest can be enough to raise clinical suspicion which leads to either a CT and MRI which will be able to characterize the lesion with its large, expansile characteristic along with fluid-filled levels. If such a mass is detected on imaging, the next appropriate step would be image-guided biopsy of the target and confirm with histopathology.

The diagnosis of ABC can often be missed if the clinician involved does not include it in the differential diagnosis. Low back pain is an extremely common complaint and a leading cause of visits to emergency departments, walk-in clinics, and family physician visits.^8^ It is a non-specific symptom that can often continue for a long period of time before further workup is done. This case outlines the importance of being aware of the potential insidious and non-specific presentation ABC can have and to be cognizant of the radiographic characteristics that suggest a destructive process.

There are various treatments for ABC including both surgical and non-surgical options. Different approaches include curettage, excision, selective arterial embolization or radiotherapy. The decision to treat and which options are most feasible depend on patient preferences, the location of the mass and its size.^4^ However, among patients who undergo curettage or surgical resection, recurrence rates are high and vary from 10 to 59%.^9^ These lesions tend to recur more often in pediatric patients (especially under 15 years old).^9,10^ Treatment with radiotherapy yields lower recurrence rates but has been associated with radiation-induced morbidity and is therefore less common.^4^ Percutaneous doxycycline has also been documented to have favorable response for the treatment of ABCs.^11^ A recent study has demonstrated favorable clinical and imaging regression of disease with a single injection of doxycycline and could be considered as a novel therapy for management of ABCs.^11^

In recent years, Denosumab has been used to successfully treat ABCs with the aim to reduce tumor size.^12^ Denosumab has traditionally been used to treat giant cell tumor of bones, with which ABCs share some pathological features, and are now being used to manage ABCs in an off-label manner.^1^ In particular, the use of denosumab for spine or pelvic ABCs is especially important as surgical outcomes in those areas may lead to serious morbidity.^12^ While not yet approved by the Food and Drug Administration, several case series have been published and the existing literature suggests that Denosumab therapy is an effective therapy for ABC. The literature supports that Denosumab leads to favorable clinical and radiological response, which has also been demonstrated in the case of the patient in this case report.^1^

## Conclusion

ABCs only represent a small percentage of primary bone tumors but together with slow growing and benign or locally aggressive lesions, are important differentials to consider when presented with chronic non-specific pain. We present a case of thoracic spine ABC causing critical central canal stenosis but with no clinical neurological findings. This case also appropriately highlights the importance of an accompanying clinical history and examination as this would define a different treatment plan.

## Learning points

1. Back pain is a very common presenting complaint but can be the first and only clinical sign to indicate severe underlying pathologies.
2. Subtle and vague longstanding ‘musculoskeletal pain’ can be explained by chronically slow growing benign but locally aggressive tumors such as an ABC.
3. Importance of multimodality imaging in obtaining and narrowing a diagnosis. CT aided in assessing bone destruction. MRI characterized the internal structure of the lesion and extent of local involvement. Fluid–fluid levels point towards a vascular lesion such as an ABC, giant cell tumor and also seen in aggressive lesions such as telangiectatic osteosarcoma. Ultrasound-guided biopsy obtained the diagnosis.
4. Critical central canal stenosis noted on imaging may be present in neurological asymptomatic patients. This highlights the importance of marrying clinical findings with radiological findings. This is critical in deciding the appropriate treatment plan for the patient.
